# The influence of air temperature on the subjective feelings of barrier suit users

**DOI:** 10.1007/s00484-025-02987-4

**Published:** 2025-07-22

**Authors:** Magdalena Młynarczyk, Aleksandra Kopyt, Katarzyna Lindner-Cendrowska, Anna Mróz, Magdalena Warszewska-Makuch

**Affiliations:** 1 Laboratory of Thermal Load, Department of Ergonomics, Central Institute for Labour Protection– National Research Institute, Warsaw, Poland; 2 Climate Research Department, Institute of Geography and Spatial Organization, Polish Academy of Sciences, Warsaw, Poland; 3 Faculty of Physical Education, Józef Piłsudski University of Physical Education, Warsaw, Poland; 4 Laboratory of Social Psychology, Department of Ergonomics, Central Institute for Labour Protection– National Research Institute, Warsaw, Poland

**Keywords:** Thermal comfort, Barrier suit, Covid-19, PPE

## Abstract

A set of protective clothing against infectious agents (PPE) is intended to prevent infection with pathogens, and the required high level of protection hinders heat exchange due to sweat evaporation. In heat stress conditions, evaporative heat loss through the skin from the user body to the external environment is then difficult or very limited, resulting in a significant impairment of overall heat exchange/transfer and, consequently, affects the productivity and health of PPE users. In order to check how the type of PPE clothing used and particular microclimatic conditions affect the subjective feelings of users, tests were conducted under controlled conditions in a climatic chamber. Two variants of the study were conducted: W1 - set with a barrier suit at an air temperature of 29 ^o^C, W2 - set with a barrier suit at an air temperature of 22 ^o^C. The results of the conducted studies indicate that the temperature of conducting the test has an impact on the subjective assessments of users of barrier clothing, after just 1 h of exposure. Controlling the air temperature (e.g. in a room) through air conditioning can reduce the intensity of physiological and psychomotor disorders.

## Introduction

Protective clothing can be characterized by thermal parameters such as water vapor resistance (R_et_) and thermal insulation (R_ct_). Both parameters affect the microclimate under clothing and the heat exchange between humans and the external environment. The value of water vapor resistance affects the comfort of using clothing (Maklewska 2010). The highest comfort of use is provided by clothing made of a material package with the lowest R_et_ coefficient. A low R_et_ value facilitates the evaporation of secreted sweat.

A set of protective clothing against infectious agents (PPE) is intended to prevent infection with pathogens, and the required high level of protection hinders heat exchange due to sweat evaporation. In heat stress conditions, evaporative heat loss through the skin from the user body to the external environment is then difficult or very limited, resulting in a significant impairment of overall heat exchange/transfer and, consequently, affects the productivity and health of PPE users (Bose-O’Reilly et al. 2021; Chen et al. 2020; Davey et al. 2021).

To protect against SARS-CoV-2, medical services most often used barrier suits. These products are most often made of materials that do not allow water vapor to pass through, to ensure the required level of protective properties. Long-term work in a protective suit is a particular burden on the body, which, while protecting the entire body from infectious agents, also constitutes a barrier to heat exchange between the body and the environment. Additionally, the need to use, as well as the type of protective clothing, depends on the established level of risk of infectious agents penetrating the skin (which may be damaged) and transferring infectious agents to other people or to other places.

The results of a study by Hunt et al. (2023) conducted among 59 healthcare workers in an emergency hospital (Australia) showed that 97% of respondents reported experiencing at least one heat-strain symptom. Thirst, warmth, fatigue, and irritability were commonly reported (> 60%). More than 50% reported mild or severe dizziness or headache, and approximately 30% reported mild or severe dizziness, faintness, or weakness (Hunt et al. 2023).

Not only does barrier clothing affect its wearer, but microclimatic conditions also affect human thermal comfort, well-being, mental and physical condition and the health of people staying in it. Disturbance of thermal balance translates into the feeling of thermal discomfort. It should be noted that the sensation of thermal comfort is important, not only for physiological and psychological reasons, but also from the point of view of psychomotor skills. “Knocking out” of the state of balance causes a feeling of dissatisfaction, impairing their attentional focus, decision making and problem-solving abilities, an increase in the number of mistakes made and a deterioration of psychomotor functions (Davey et al. 2021; Gwóźdź 2015; Młynarczyk and Orysiak 2024).

Moreover, rising global temperature is intensifying heat stress for workers worldwide, putting their safety increasingly under threat (Falarz et al. 2021), also for healthcare workers wearing PPE (Hunt et al. 2023; Ippolito et al. 2021; Messeri et al. 2021; Tabah et al. 2020; Unoki et al. 2020). Warm and humid conditions directly strain human body, not only leading to work capacity reduction, but also increasing the risk of heat-related illnesses (Habibi et al. 2024).

Studies conducted during COVID-19 and shortly after the pandemic provide crucial insight into working conditions, in extreme situation, often preventing adequate thermal comfort for medical staff and patients. This is still an important aspect and therefore it is important to monitor the current working situation of medical workers using barrier clothing (Młynarczyk et al. 2024). Studies of human responses, which are a very valuable source of knowledge about organism reactions to various working conditions, are in the minority compared to equally interesting survey studies. Data collected from volunteers conducted another building block of knowledge about i.a. their subjective feelings occurring depending on working conditions. Further research on physiological workload or subjective feelings are warranted as effective management practices in health care may be required to avoid excessive workload and impaired work performance.

In order to check how the type of PPE clothing (indicated as the most frequently used by paramedics) used and particular microclimatic conditions affect the subjective feelings of users, tests were conducted under controlled conditions in a climatic chamber.

## Research material

**Fig. 1:**
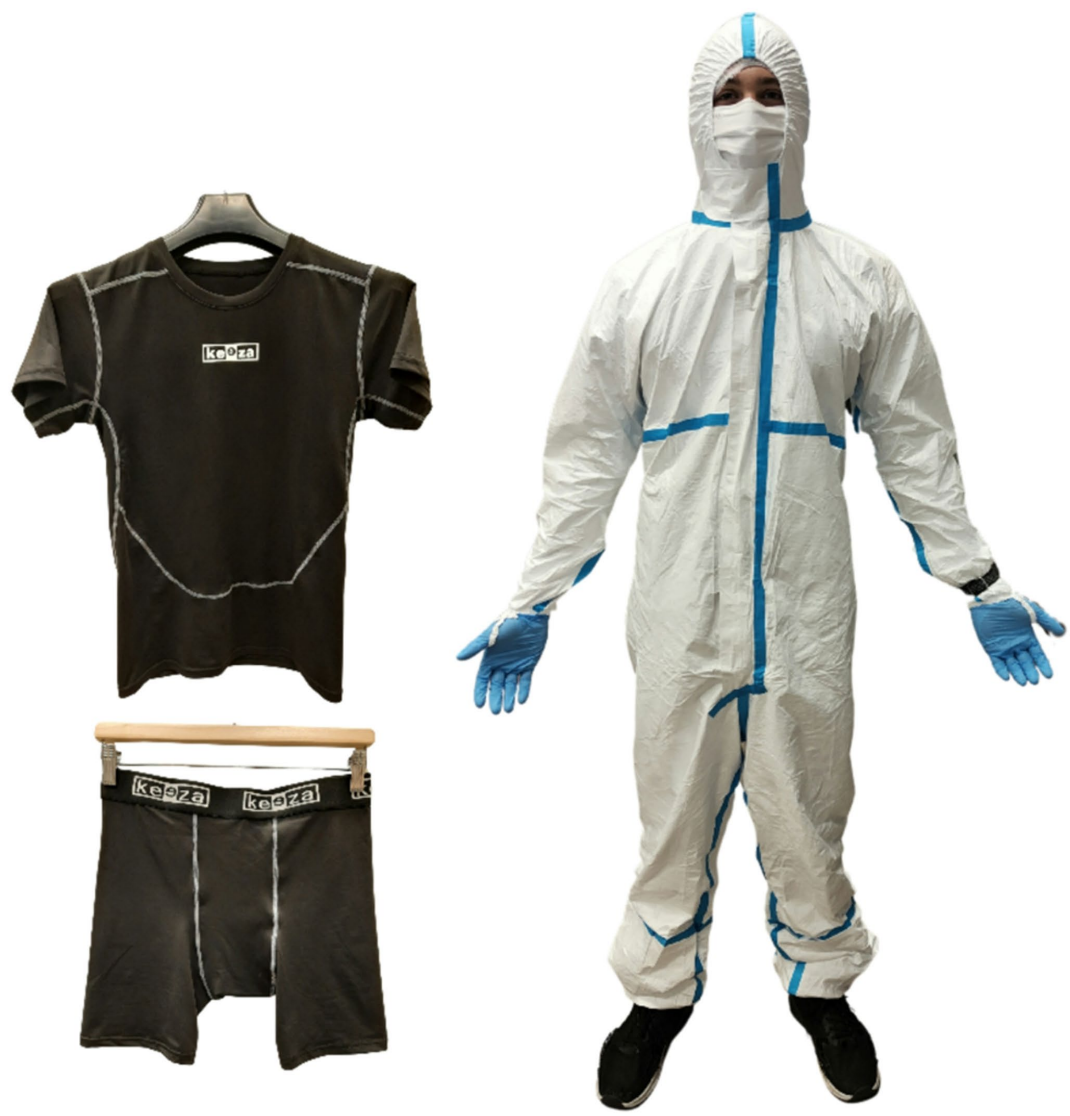
The tested clothing set - thermal underwear and Tyvek 600 coverall

The Tyvek 600 suit with covered seams, which met the requirements of the standards (EN 1073-2 (n.d.); EN 1149-5 (n.d.); EN 13034 (n.d.); EN 14126 (n.d.); EN ISO 13982 (2010), was selected for the study. Short thermal underwear (100% polyester) was worn under the suit (after consultation with the representatives of the paramedics) (Fig. 1). Additionally, the volunteers put on cotton socks, nitrile gloves, a polypropylene hygiene cap and an FFP3 filtering half-mask (Fig. 1). The volunteers worn their own sport shoes.

## Volunteers

The recruited group of 15 male students took part in the qualifying tests - physical capacity tests. From this group, 10 volunteers were selected for tests in the climatic chamber. All tests were approved by the Ethics Committee (approval no. KEBN-23-82-MM) and were conducted under medical supervision.

The volunteers first underwent body composition analysis (Tanita Body Composition Analyzer BC-418), followed by medical interview. After being admitted to the study, each volunteer performed a physical capacity test to the point of voluntary exhaustion. The qualifying tests were conducted using a horizontal ergometer (treadmill) and the CORTEX Ergospirometer, according to the protocol: 2 min of rest, 5 min of warm-up (treadmill speed 6 km/h), then an increase in treadmill speed by 2 km/h every 2 min of effort (until refusal) and 3 min of rest - treadmill speed 6 km/h. During the physical capacity test, the following parameters were analyzed: maximal oxygen consumption (VO2max), oxygen pulse (VO2max/HR), maximum heart rate (HRmax), ventilation equivalent for oxygen (VE/VO2), ventilation equivalent for carbon dioxide (VE/VCO2) and respiratory exchange rate (RER = VCO2/VO2). Based on the results of the qualification tests, 10 volunteers with the most similar physical capacity were selected. The characteristics of the study group are described in Table 1.

**Table 1:** Mean values ​​(+ SD) of parameters describing the physical capacity of the study group [oxygen uptake (VO2max), oxygen uptake per unit of body mass (VO2max/kg), age and BMI (Body mass Index)]

Parameters	mean	sd
VO2max/kg [ml/min/kg]	59.2	3.8
VO2max [l/min]	4.4	0.5
Age [years]	22	2
BMI	23	2

## Research procedure

The research was conducted according to the methodology approved by the Ethics Committee (approval no. KEBN-23-82-MM), which was intended to simulate the work of paramedics.

In order to check the influence of external temperature on subjective feelings of volunteers dressed in a barrier suit, 2 variants of tests were performed: (W1) at high temperature of 29 ^o^C and (W2) at thermoneutral temperature of 22 ^o^C (RH 40–60%, V 0.3–0.4 m/s). The tests of the above variants were conducted in random order.

The research was conducted according to the following scheme:

1. Preparation for testing in the climatic chamber:

- psychological tests: reaction time measurement, general well-being scales.
- measurement of parameters: blood pressure, ear canal temperature (control, without analysis).
- putting on tested clothes.

2) Tests in the climatic chamber:

- 65 min of exposure (physical exertion) to a simulated thermal environment.

3) Actions after completion of the test:

- measurement of parameters: blood pressure and temperature in the auditory canal (control, without analysis).
- psychological tests: reaction time measurement, general well-being scales.

During the tests in the climatic chamber, volunteers performed the activities according to the protocol described in Table 2.

**Table 2:** List of activities performed by the participant in the chamber during the study, along with their duration

No.	Time	Action
1	0–5 min	Rest in standing position on the treadmill
2	5–10 min	Warm-up: walking treadmill speed 3 km/h
3	10–20 min	Walking at a treadmill speed of 6 km/h
4	20–25 min	Resuscitation – CPR manikin: at a rate of 100 compressions per minute
5	25–35 min	Walking at a treadmill speed of 6 km/h
6	35–40 min	Resuscitation - CPR manikin: at a rate of 100 compressions per minute
7	40–50 min	Walking at a treadmill speed of 6 km/h
8	50–55 min	Resuscitation - CPR manikin: at a rate of 100 compressions per minute
9	55–60 min	Walking at a treadmill speed of 6 km/h
10	60–65 min	Rest in standing position on the treadmill

Before the study and after each of the above-mentioned stages, volunteers were asked to report their subjective perceptions of: thermal comfort, skin and clothing moisture, and the difficulty of the work performed.

The following subjective rating scales were used to assess the above-mentioned perceptions:

1. thermal sensations – ASHRAE scale – 7-point scale of thermal sensations: −3 (cold), −2 (cool), −1 (slightly cool), 0 (neutral), + 1 (slightly warm), + 2 (warm), + 3 (hot) (ANSI/ASHRAE Standard 55 (n.d.); Zwolińska and Bogdan 2013),
2. skin wettedness – Nielsen’s scale – 8-point scale of skin moisture sensations from 1 – drier than normal to 8 – sweat drips in many places (Nielsen et al. 1987; Zwolińska and Bogdan 2013),
3. clothing moisture – Nielsen’s scale – 4-point scale of clothing humidity sensation from 1 – dry to 4 – wet (Nielsen et al. 1987; Zwolińska and Bogdan 2013),
4. effort severity – the Borg Rating of Perceived Exertion (Borg RPE Scale^®^) scale (from a rating of 6—“no exertion at all” to 20—“maximal exertion”) (Borg 1970, 1998),
5. assessment of general well-being – based on selected Grandjean’s scales (Baschera and Grandjean 1979).

The Grandjean’s scale is used as a subjective measure of fatigue resulting from performing various types of work and professions and is an indicator of changes in the level of psychophysical fitness and the level of arousal, as well as an indicator of mental load resulting from performing tasks related to mental activity (Baschera and Grandjean 1979). The following subscales were used for the study: positive – negative mood, strong – weak, rested – tired, energetic – sluggish, able – unable to concentrate.

As a part of the research, before and after exposure to the conditions in the chamber and the clothing used, the speed, adequacy and uniformity of the reaction to light and sound stimuli were checked using a reaction time meter (MCZR/ATB 1.0).

## Research results

The analysis of subjective feelings was performed by calculating the average values ​​obtained from individual responses. Below is an analysis of the results of subjective feelings divided into study variants.

### Thermal comfort

**Fig. 2:**
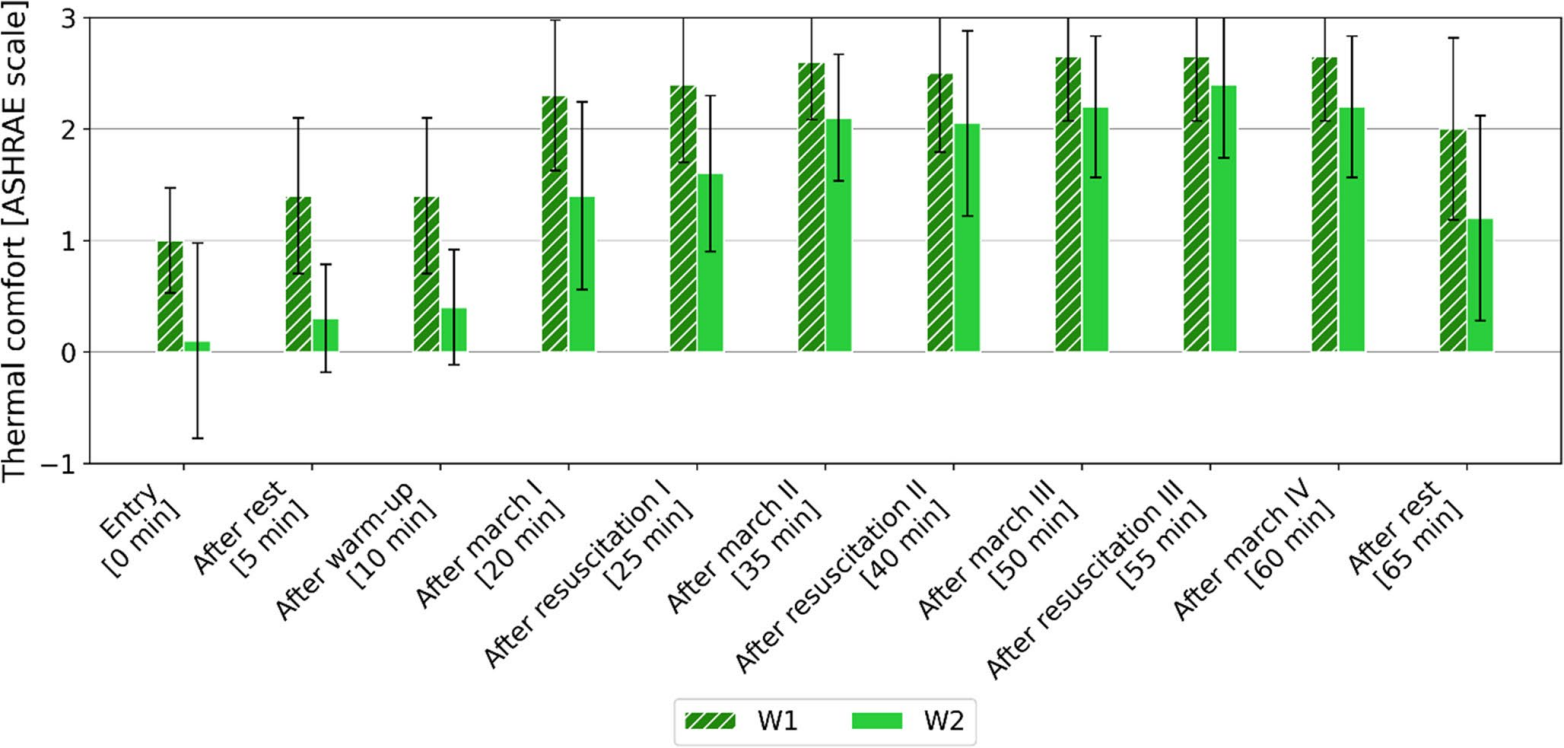
Change in the mean value of thermal comfort (± SD) assessed by volunteers according to the ASHRAE scale [−3 (cold), −2 (cool), −1 (slightly cool), 0 (neutral), + 1 (slightly warm), + 2 (warm), + to + 3 (hot)], depending on the study variant

The 7-point ASHRAE scale (from − 3 “cold” to + 3 “hot”) was used to assess the thermal comfort of the whole body. The obtained mean values ​​with standard deviation are presented in Fig. 2.

Based on the obtained average values ​​of thermal comfort, it can be stated that the air temperature (and activities) during the study has a greater influence on subjects’ perception than the clothing used. In the extended range of feeling (−1 to + 1), thermal comfort was felt by volunteers only during studies conducted at a temperature of 22 ^o^C and only for the first 10 min of the trial. After the first 10-minute walk, the value of the thermal sensation index was already about 1.5 (feeling between"slightly warm” and “warm”). In the case of studies at 29 ^o^C, at the very beginning of the study while entering the climatic chamber, volunteers assessed their thermal sensations as"slightly warm” (> 1), but with time and rising intensity of their activity, volunteers perceived thermal conditions as mostly “warm” and “hot” (TSV equal + 2 or + 3), regardless of the type of activity (resuscitation or marching).

### Skin wettedness

**Fig. 3:**
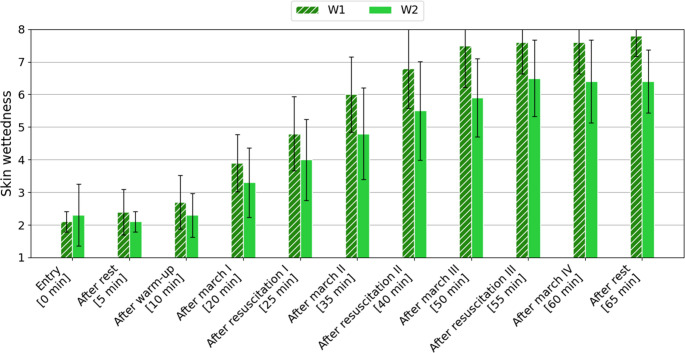
Change in mean skin wettedness (± SD), assessed by volunteers according to the Nielsen’s scale, depending on the study variant [1 (drier than normal), 2 (normally dry), 3 (some body parts moist), 4 (larger body parts moist), 5 (some body parts wet), 6 (larger body parts wet), 7 (sweat drips in some places), 8 (sweat drips in many places)]

The skin wettedness was assessed using an 8-point Nielsen’s scale (from 1 “drier than normal” to 8 “sweat drips in many places”). The mean values ​​obtained with standard deviation are presented in Fig. 3.

In the case of skin wettedness, for the first 10 min of the study, regardless of the variant, the volunteers assessed their skin as “normally dry”. After the first 10-minute treadmill walk, higher subjective assessment values ​​were recorded for variant W1. At an air temperature of 29 ^o^C, when using a barrier suit, higher skin moisture assessment values ​​were recorded.

### Clothing moisture

**Fig. 4:**
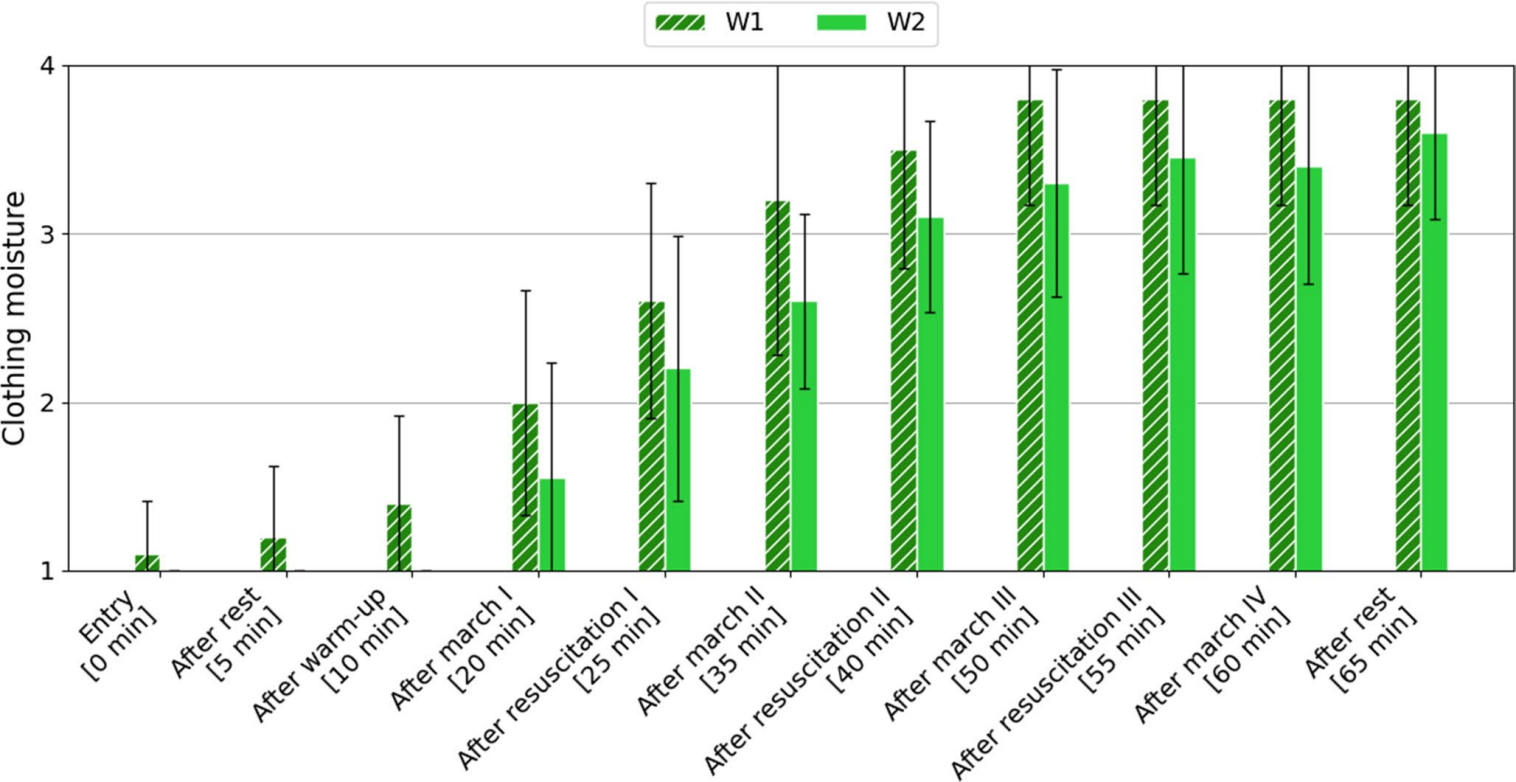
Change in mean clothing moisture content (± SD), assessed by volunteers according to the Nielsen’s scale, depending on the study variant [1 (dry), 2 (slightly moist), 3 (moist), 4 (wet)]

The 4-point Nielsen’s scale (from 1 “dry” to 4 “wet”) was used to assess clothing moisture content. The mean values ​​obtained along with the standard deviation are presented in Fig. 4.

In the case of clothing moisture, the highest values ​​throughout the study were recorded for variant W1. After 50 min of the study, volunteers in variant W1 rated the clothing as 4 (“wet”), while in variant W2 it remained at the same level of about 3.5 (between “moist” and “wet”).

### The difficulty of the work performed

**Fig. 5:**
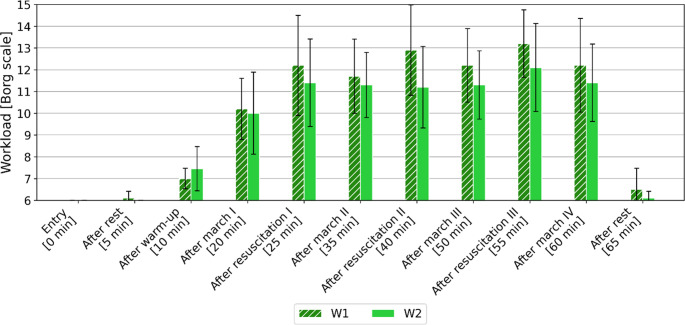
Change in mean exercise intensity (± SD) assessed by volunteers according to the Borg scale, depending on the study variant

The Borg’s scale was used to assess the difficulty of the work performed (from 6 “no effort” to 20 “maximum effort”). The obtained mean values ​​with standard deviation are presented in Fig. 5.

The intensity of the effort performed during the tasks performed by the volunteers was assessed as follows:

- warm-up – as “exceptionally light” (approx. 7) (regardless of the test variant).
- first treadmill walk – as “light” (approx. 10) (regardless of the test variant).
- first resuscitation – as “moderate” for W1 (approx. 12) and as “light” for W2.
- second treadmill walk – as “light” (approx. 11) (regardless of the test variant).
- second resuscitation – as “quite heavy” for W1 (approx. 13), and as “light” for W2 (approx. 11).
- third treadmill walk – as “moderate” for W1 (approx. 12) and as “light” for W2 (approx. 11).
- third resuscitation – as “quite severe” for W1 (approx. 13), as “moderate” for W2 (approx. 12).

It should also be noted that after each resuscitation in the case of variant W1, the volunteers assessed such an activity as a more difficult effort in relation to variant W2. Although the group of volunteers was characterized by good physical capacity, performing resuscitation was assessed as a rather difficult effort.

### Overall well-being assessment

The general assessment of well-being among the volunteers was conducted using selected Grandjean’s scales.

**Fig. 6:**
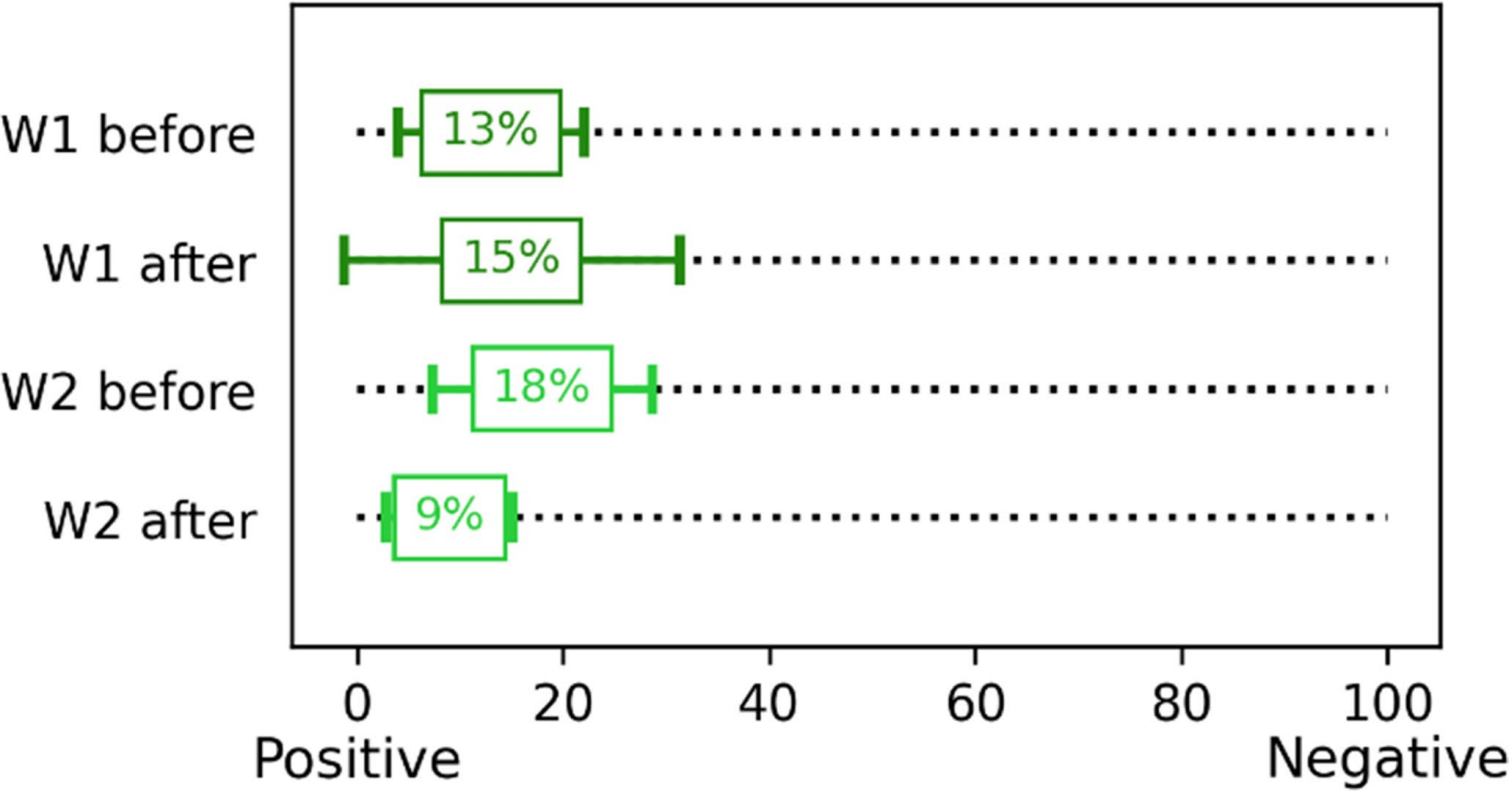
Volunteers’ responses to the question “What is your mood at the moment?” before and after exposure in the climatic chamber (mean value with standard deviation)

Before and after the test in the chamber, the volunteers answered the question: “What is your mood at the moment?” on a scale from 0 (“positive”) to 100 (“negative”). After the tests for the W2 variant, the volunteers felt more positive than before the test. In the case of the W1 variant, a slight decrease in mood in the negative direction was observed after the test (Fig. 6).

In response to the statement “I feel now” (Table 33) on a strong-weak scale, a slight decrease in well-being towards “weak” was observed in the volunteers in the case of variant W1 after the test. In the case of variant W2, no changes were observed (Table 3). In response to the question regarding well-being on a rested-tired scale, a decrease in feelings towards “tired” was observed in the volunteers in the case of variant W1 after the test by 9%, and in the case of variant W2, a decrease of 2% was observed (Table 3). In response to the question “I feel now: on an energetic-sluggish scale”, a slight decrease in well-being towards “sluggish” was observed in the volunteers in the case of variant W1 after the test. In the case of variant W2, an improvement in well-being towards “energetic” was noted (Table 3). In response to the question regarding the ability to concentrate, a deterioration towards “unable to concentrate” was noted in the volunteers in the case of variant W1, an improvement towards “able to concentrate” was noted in the case of variant W2 (Table 3).

**Table 3:**
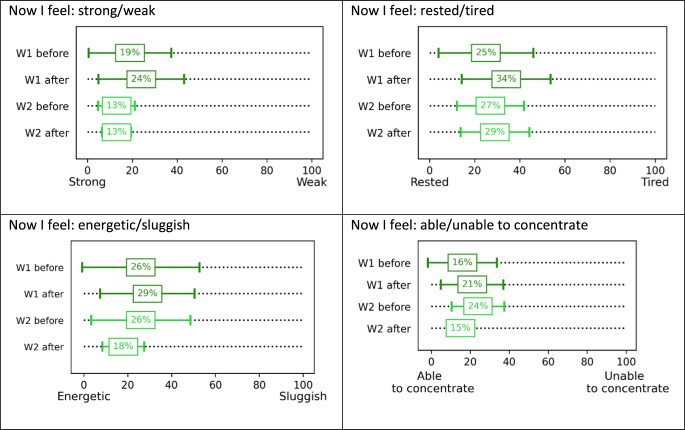
Volunteers’ responses to the question “how do you feel now” before and after exposure in the Climatic chamber (mean value with standard deviation)

The results of comparisons of the next two measurements of the Grandjean’s scale in variant W1 indicate their changes towards an increased feeling of fatigue, a decreased ability to concentrate, a decreased strength and an increased feeling of exhaustion. In variant W2, on the other hand, changes were noted towards an increased feeling of fatigue, as well as an increased energy and an increased ability to concentrate.

### Response time

The reaction time meter (MCZR/ATB 1.0) measured eye-hand coordination, concentration and reaction speed. The study used a 120-second program that contained 22 stimuli (including double reactions).

As part of the reaction time study, the following parameters were measured and analyzed:

- mean reaction time (tM),
- minimum reaction time (tL),
- maximum reaction time (tH),
- number of correct responses (COR),
- number of incorrect no-responses (wNR),
- number of correct no-responses (cNR),
- number of reactions with the wrong button (wB),
- number of reactions when no reaction was required (UR), and.
- number of reactions between stimuli (RBS).

**Fig. 7:**
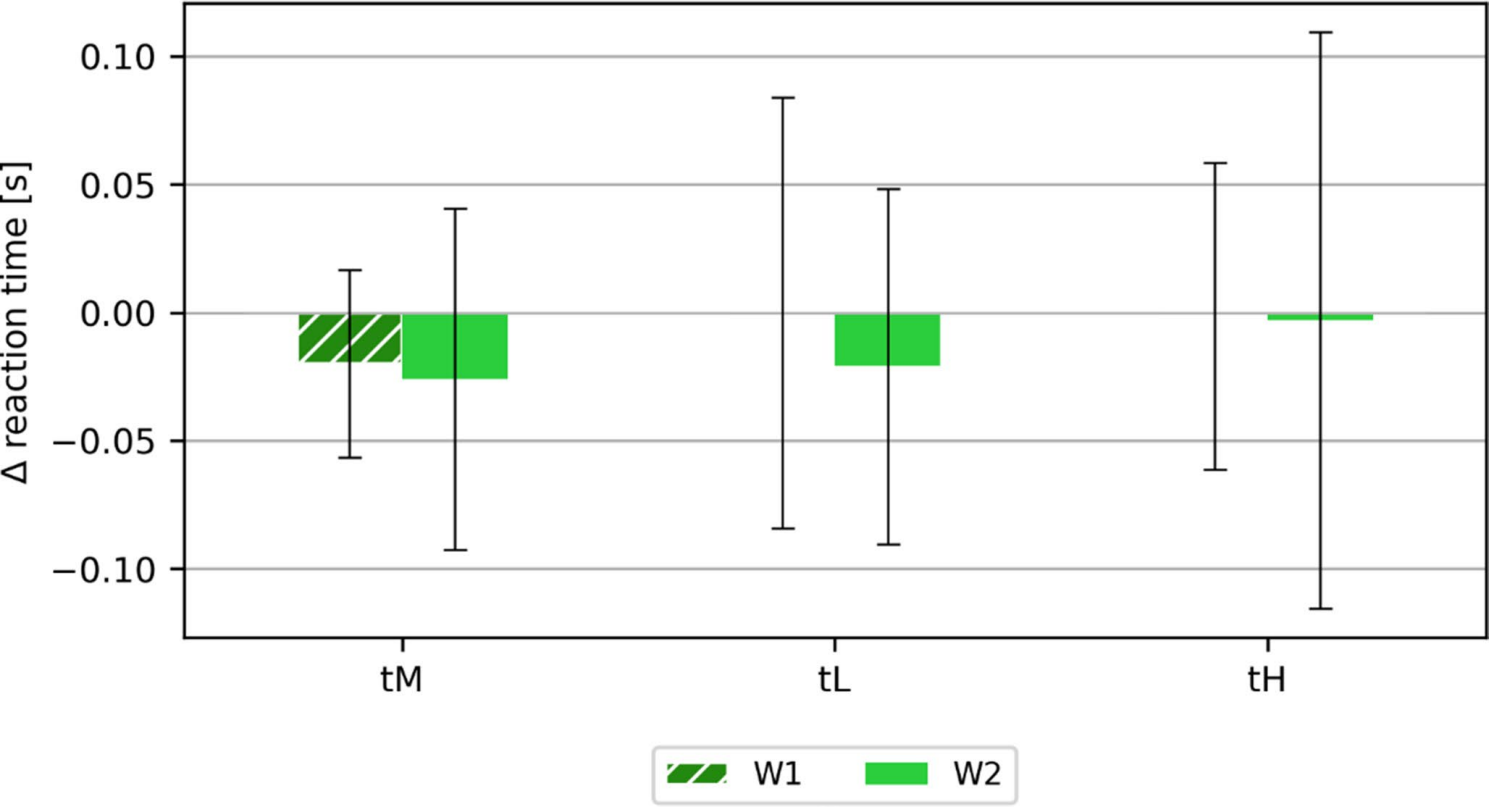
Calculated mean differences (± SD) after and before the test for the categories: mean reaction time (tM), minimum reaction time (tL), maximum reaction time (tH)

As part of the analysis of the results, the difference between the values ​​obtained after and before the test was calculated. Figure 7 shows the calculated difference of the average reaction time (tM), the minimum reaction time (tL) and the maximum reaction time (tH).

There were no significant differences in reaction time (average approx. 0.6 s, minimum/shortest approx. 0.4 s, maximum/longest approx. 0.9 s). The values ​​obtained were within their standard deviation ranges. However, in the case of the average reaction time, it was slightly shorter after the tests (regardless of the test variant).

**Fig. 8:**
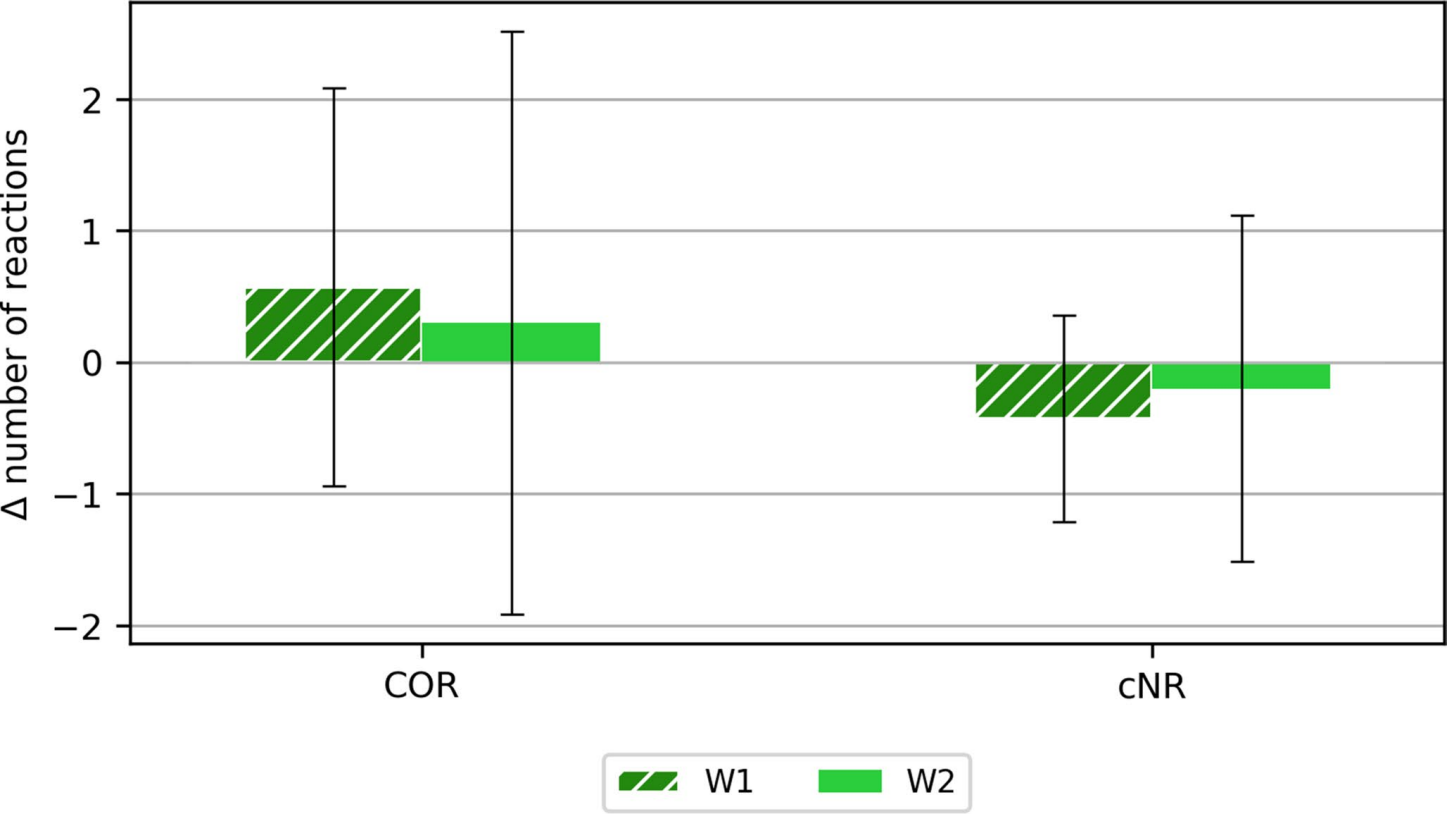
Calculated mean differences (±SD) after and before the main study for the categories: correct responses (COR), correct non-responses (cNR)

**Fig. 9:**
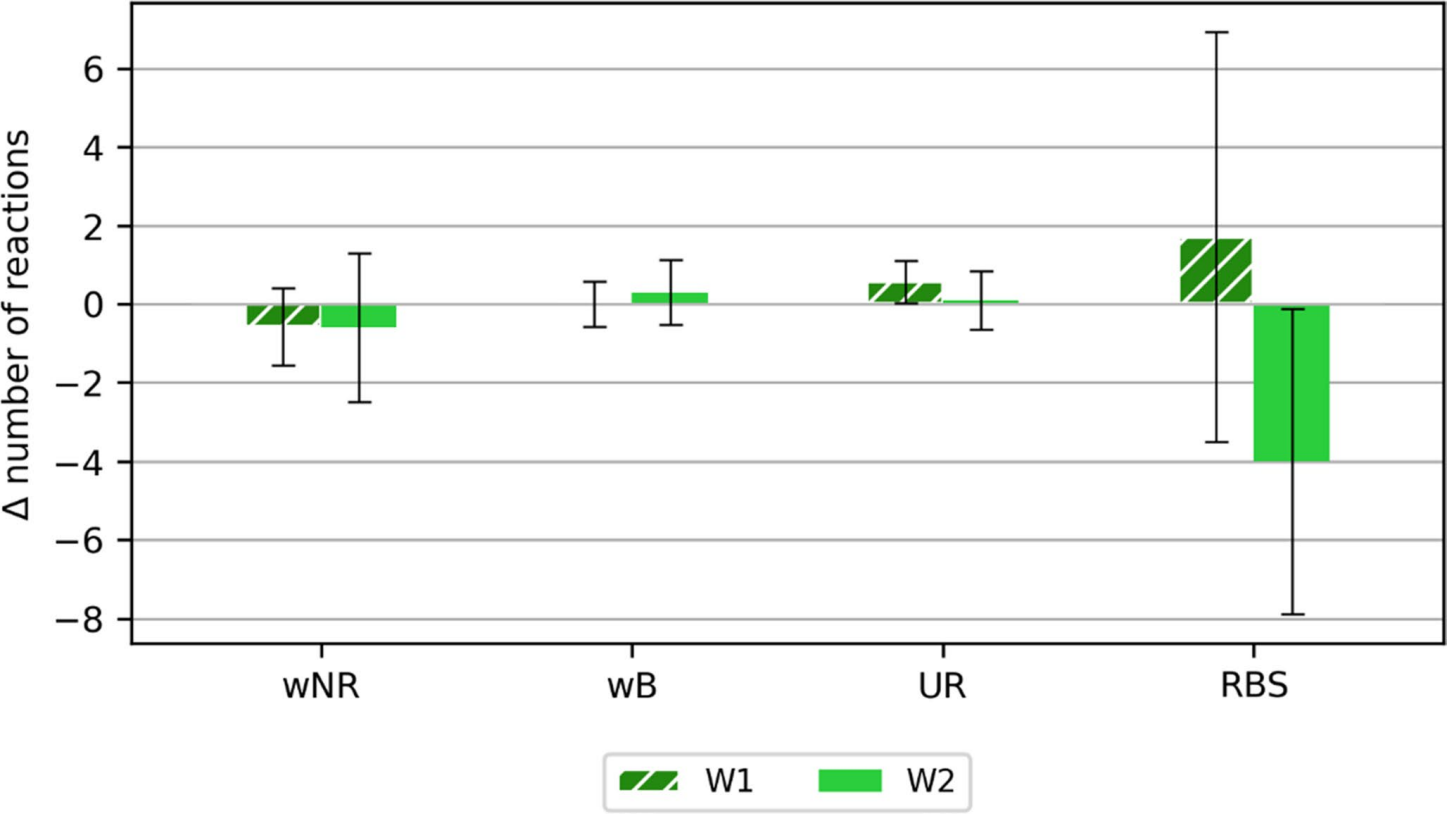
Calculated mean differences (±SD) after and before the main test for the following categories: incorrect no response (wNR), incorrect button response (wB), response when no response was required (UR), response between stimuli (RBS)

Figures 8 and 9 show the differences in the numbers of responses after and before the exercise in the chamber for the following categories: correct responses (COR), incorrect no responses (wNR), correct no responses (cNR), wrong button responses (wB), responses when no response was expected (UR) and responses between stimuli (RBS).

The greatest differences obtained with the reaction time measure were noted for the reaction between stimuli (RBS). The greatest differentiation - standard deviation was also noted for this parameter. More reactions between stimuli were noted after the study for variant W1, while in the case of variant W2, more reactions between stimuli were noted before exposure in the chamber. Perhaps a longer exposure to high temperature (and/or a less trained group of volunteers) would have contributed to greater variability in responses when using a reaction time measure.

## Summary and discussion

Two variants of the study were conducted: W1 - set with a barrier suit at an air temperature of 29 ^o^C, W2 - set with a barrier suit at an air temperature of 22 ^o^C. As part of the study in the climatic chamber, volunteers were asked to subjectively assess their feelings in the following areas: thermal comfort of the whole body, moisture of the skin and clothing, as well as the difficulty of the work performed. Before and after exposure in the chamber, tests of reaction time were also conducted, and a subjective assessment of general well-being was conducted. The obtained results confirmed the influence of temperature on the subjective feelings of barrier suit users. The subjective feelings in the workplace are an important element and translate into the efficiency and safety of the work performed (Młynarczyk and Orysiak 2024; Młynarczyk et al. 2024).

The use of personal protective equipment (PPE) including gloves, respiratory protection, and an impermeable gown or coverall impairs the body’s ability to regulate core temperature and exposes personnel to a greater risk of fluid loss (dehydration), overheating (hyperthermia), and reduced performance, potentially resulting in accidental illness (Figs et al. 2023). A survey of 2,191 hospital workers in China showed that most healthcare workers experienced heat stress. Before putting on PPE, most healthcare workers felt “warm (+ 2)” (55.7%) or “hot (+ 3)” (30.2%). However, after putting on PPE and completing their activities, the percentage of “hot (+ 3)” responses increased to 70.1% (for “warm (+ 2)” − 26.7%). In the presented studies, in the case of thermal comfort, a difference in subjective feelings was noted at the very beginning of the study (from entering the chamber, through rest and light physical exertion). After about 20 min of exposure, volunteers in W2 felt the environment as “quite warm” (+ 1), while in variant W1 the sensation was “warm” (> + 2). Such a value for W2 was not noted even after 60 min of exposure. The obtained results do not indicate that the type of physical activity influenced thermal perception (e.g. “resuscitation” vs. “marching”), although according to the Borg’s scale, resuscitation was assessed as a more intense effort, especially in variant W1.

According to the literature, as a result of wearing PPE, thermal sensations significantly worsened (towards maximum thermal discomfort) (Zhu et al. 2023), which was also demonstrated in this study after just 1 h of exposure in barrier clothing.

Long-term use of PPE can cause dizziness, thermal discomfort and headaches (Zhao et al. 2023), mostly due to dehydration and overheating of the body. In the study conducted by Zhao et al. (2023), workers collecting genetic material complained of: profuse sweating, dehydration, heat cramps and even heat stroke. Similar feelings were indicated by 224 respondents of a survey conducted in the UK (Davey et al. 2021). They indicated that after using PPE, almost 60% had problems with concentrating, solving complex problems (27%), making decisions (22%) and with short-term memory (20%) (Davey et al. 2021). In the presented studies, the workload was significantly higher (than collecting genetic material), and despite this, volunteers did not report such drastic symptoms. In the presented studies for W1 and W2, in the case of skin moisture, a difference of up to 2 points on the scale was noted between the variants. The sensations of clothing humidity were similar, only differences within 0.5 points of the scale were noted at the end of the study to the detriment of variant W1. It should be noted, however, that this was only a 1-hour exposure. In addition, the general assessment of well-being also showed a deterioration in the states of sensation after exposure to a higher temperature. For W1, for subjective assessments using the Grandjean’s scale, changes were observed towards an increase in the feeling of fatigue, a decrease in the ability to concentrate, a decrease in strength and an increase in the feeling of exhaustion.

Whereas, the feeling of discomfort and deterioration of well-being may affect the correctness of the tasks performed (Saini et al. 2017; Rastegar et al. 2021). Surveys conducted among 505 nurses in Turkey indicated the influence of protective clothing (PPE) on the number of medical errors (Cennet et al. 2023). The largest number of errors made by nurses using PPE were related to: needle/sharp instrument injuries (47%), hospital infections (39%), pressure sores in patients (28%), falls (23%) and administration of the wrong dose of medication (17%).

Not only the PPE used but also the temperature can influence the human body’s response. In presented studies, the severity of the activities performed was also assessed to be greater for the variant of the study conducted at a higher temperature (W1).

The results of the conducted studies indicate that the temperature of conducting the test has an impact on the subjective assessments of users of barrier clothing, after just 1 h of exposure. Controlling the air temperature (e.g., in a room) through air conditioning can reduce the intensity of physiological and psychomotor disorders. In the case of work performed in personal protective equipment in the field/in the open space (e.g., pre-hospital care, transport of patients from emergency medical services to hospital, external centres), it is worth ensuring cooler conditions there, either through local ventilation or other technological solutions.

## Data Availability

The datasets analysed during the current study are available from the corresponding author on reasonable request.
